# Mechanical Stability of Ribonuclease A Heavily Depends on the Redox Environment

**DOI:** 10.1021/acs.jpcb.2c04718

**Published:** 2022-08-17

**Authors:** Pamela Smardz, Adam K. Sieradzan, Paweł Krupa

**Affiliations:** † Institute of Physics, Polish Academy of Sciences, Al. Lotników 32/46, 02-668 Warsaw, Poland; ‡ Faculty of Chemistry, University of Gdańsk, Wita Stwosza 63, 80-308 Gdańsk, Poland

## Abstract

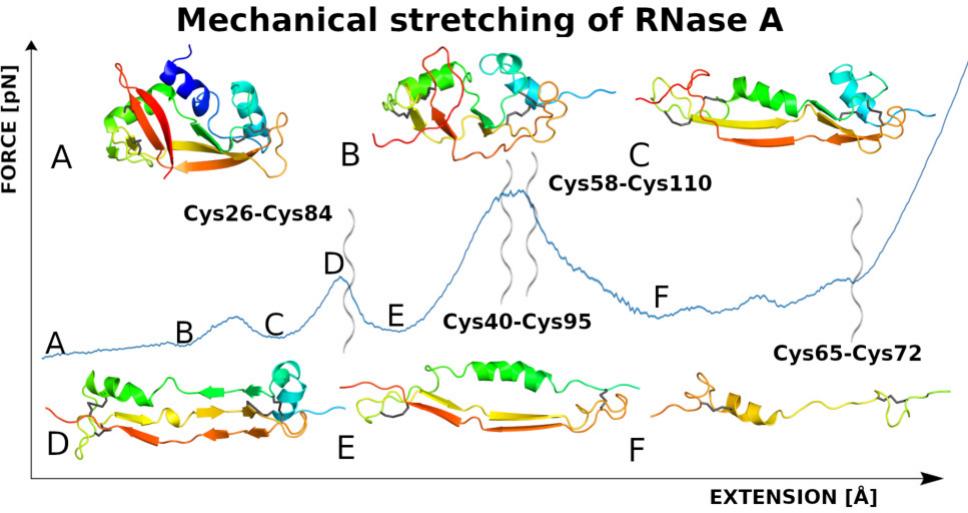

Disulfide bonds are covalent bonds that connect nonlocal fragments of proteins, and they are unique post-translational modifications of proteins. They require the oxidizing environment to be stable, which occurs for example during oxidative stress; however, in a cell the reductive environment is maintained, lowering their stability. Despite many years of research on disulfide bonds, their role in the protein life cycle is not fully understood and seems to strictly depend on a system or process in which they are involved. In this article, coarse-grained UNited RESidue (UNRES), and all-atom Assisted Model Building with Energy Refinement (AMBER) force fields were applied to run a series of steered molecular dynamics (SMD) simulations of one of the most studied, but still not fully understood, proteins—ribonuclease A (RNase A). SMD simulations were performed to study the mechanical stability of RNase A in different oxidative–reductive environments. As disulfide bonds (and any other covalent bonds) cannot break/form in any classical all-atom force field, we applied additional restraints between sulfur atoms of reduced cysteines which were able to mimic the breaking of the disulfide bonds. On the other hand, the coarse-grained UNRES force field enables us to study the breaking/formation of the disulfide bonds and control the reducing/oxidizing environment owing to the presence of the designed distance/orientation-dependent potential. This study reveals that disulfide bonds have a strong influence on the mechanical stability of RNase A only in a highly oxidative environment. However, the local stability of the secondary structure seems to play a major factor in the overall stability of the protein. Both our thermal unfolding and mechanical stretching studies show that the most stable disulfide bond is Cys65–Cys72. The breaking of disulfide bonds Cys26–Cys84 and Cys58–Cys110 is associated with large force peaks. They are structural bridges, which are mostly responsible for stabilizing the RNase A conformation, while the presence of the remaining two bonds (Cys65–Cys72 and Cys40–Cys95) is most likely connected with the enzymatic activity rather than the structural stability of RNase A in the cytoplasm. Our results prove that disulfide bonds are indeed stabilizing fragments of the proteins, but their role is strongly redox environment-dependent.

## Introduction

A disulfide bond is a covalent bond formed between two cysteine residues by oxidation of thiol groups and can be of inter- or intramolecular origin. The formation of disulfide bonds is the most unique type of post-translational modification,^1,2^ and it is a necessary step for most proteins containing disulfide bonds to obtain the correct conformation.^3^ It takes place primarily in the endoplasmic reticulum (ER),^4^ but it is not limited to it, as it can occur in mitochondrial intermembrane space or bacterial periplasm.^5^

In eukaryotes, the ER has a more oxidizing environment than the cytoplasm,^6−8^ which is an important factor in thiol–disulfide exchange reactions that are governed by a variety of catalysts.^9^ Reducing–oxidizing conditions, next to p*K*_a_–pH differences, and the spatial accessibility of cysteines are important factors that influence the formation of the disulfide bonds.^10,11^

Disulfide bonds are common in nature. On average, more than 50% of cysteine residues in proteins can form a disulfide bond,^12^ and more than 20% of proteins have at least one disulfide bond^13^ present. Disulfide bonds are present in some of the proteins in reductive cytoplasm,^14^ and their number significantly increases during oxidative stress owing to reversible disulfide bond formation in these conditions.^15,16^

Although disulfide bonds are studied intensively, both experimentally^17,18^ and theoretically,^19,20^ they are still an interesting topic for the scientific community.^21−23^ Their role is yet to be understood fully because some disulfide bonds do not seem to be essential for conformational stability and dynamics (at least on short time scales),^24^ and the addition of a new disulfide bond does not always increase protein stability,^25^ while its removal does not always compromise the stability.^26^ It is known that covalent bridges, such as disulfide bonds, can play other roles such as maintaining rigidity or preventing enzymatic proteolysis while destabilizing the structure.^27^ Their presence can also lead to amyloidosis,^21^ or they can be used in regulation mechanisms.^28−30^ Recent observations show that the presence of the disulfide bonds stabilizes viral capsids^31,32^ and is crucial for the activity of many viral proteins, including SARS-COV-2.^22^

**Figure 1:**
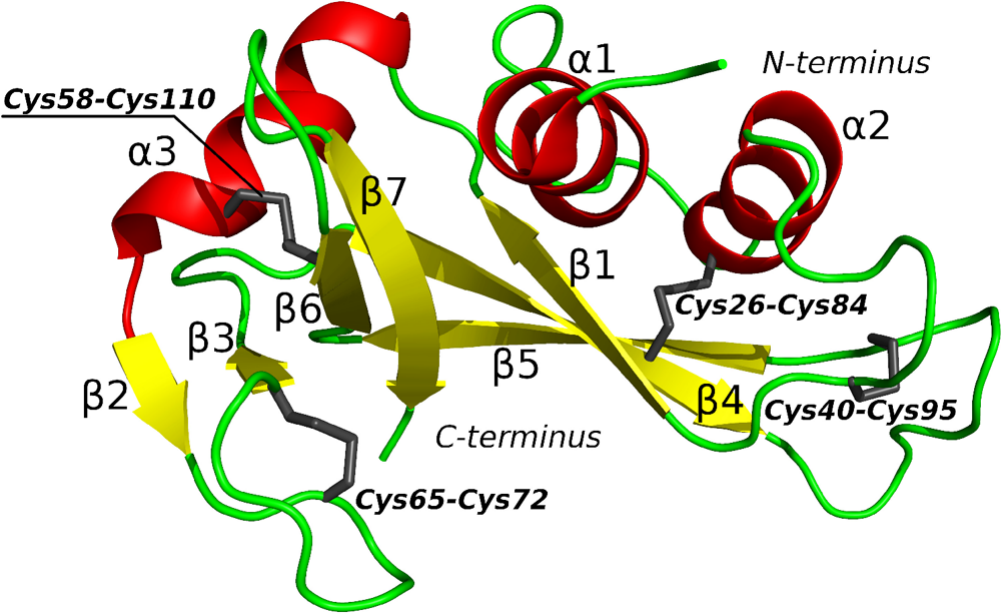
Cartoon representation of RNase A (PDB code: 1KF5) with the cysteine residues and disulfide bonds marked by black sticks. Symbols α and β indicate α-helices and β-sheets, respectively.

Ribonucleases are an exceptionally well-studied group of enzymes that contain disulfide bonds.^33−35^ Since the determination of the bovine pancreatic ribonuclease A (RNase A) amino acid sequence in 1960,^36^ a number of experiments have been performed such as thermodynamic structural studies,^37^ tyrosyl–carboxylate ion hydrogen bonding,^38^ kinetic denaturation,^39^ and much more. RNase A consists of three helices (H1: Thr3–Met13, H2: Asn24–Arg33, and H3: Ser50–Gln60) and seven β-sheets (B1: Val43–Val47, B2: Lys61–Val63, B3: Cys72–Gln74, B4: Met79–Glu86, B5: Tyr97–Lys104, B6: Ile106–Glu111, and B7: Val116–Ser123 ) and has four disulfide bonds: Cys26–Cys84, Cys40–Cys95, Cys58–Cys110, and Cys65–Cys72 (Figure 1)

The lack of even one of the disulfide bonds disrupts RNase A folding,^3^ which makes RNase A a particularly good model protein for the study of the influence of the disulfide bond on protein folding and unfolding processes.^40^ The influence of various physiological conditions, such as hydrostatic pressure^41^ and temperature, on the disulfide bond stability was studied both experimentally^42^ and theoretically.^43^ Our recent computational studies^44^ revealed the low thermal stability of the Cys40–Cys95 disulfide bond contrary to the other three disulfide bonds, which were relatively stable at all investigated temperatures.

In this article, the influence of the disulfide bond breaking/formation on the mechanical stability of RNase A was investigated by performing Steered Molecular Dynamics (SMD) simulations in all-atom Assisted Model Building with Energy Refinement (AMBER) and coarse-grained UNited RESidue (UNRES) force fields. The most semistable intermediate structures were identified, revealing the high mechanical stability of the Cys65–Cys72 disulfide bond owing to the high stability of this region, which was also observed experimentally.^45^ Moreover, the RNase A mechanical stability depends on the redox environment. In the highly oxidizing environment, RNase A is more mechanically stable.

## Methods

As only the UNRES model allows us to study the dynamic breaking and formation of the disulfide bonds in the course of simulations,^46^ including the possible formation of non-native bonds, it was selected as the primary method for this investigation. To compare results, the second set of simulations was run in the all-atom Amber model. This force field allows the study of only static disulfide bonds that are present or absent during the whole simulation. To mimic the bond breaking in the all-atom simulations, restraints were imposed on the pairs of reduced sulfur atoms of cysteine residues that natively form disulfide bonds. Performing simulations with both coarse-grained and all-atom models is beneficial, allowing deeper examination of the system on different levels of resolution and allowing self-testing of the methods.

### UNRES Coarse-Grained Model

UNRES is a physics-based coarse-grained force field developed for running simulations of peptides and proteins, in which^47−49^ the polypeptide chain is reduced to two centers of interaction per residue, namely the peptide group (p) located halfway between the two centers, consecutive C^α^ atoms and the united side-chain (SC). The geometry of the polypeptide chain is defined by C^α^ and the geometry centers of the side-chains, and the mass is uniformly distributed along the bond (as in the stick) rather than in the single point, allowing for more physical dynamics.^50^ Owing to simplification of the polypeptide chain and omission of the secondary degrees of freedom, in the UNRES coarse-grained model events occur 3–4 orders of magnitude faster than in all-atom force fields.^51^ The energy in UNRES, which is represented by a potential of mean force (PMF) of a given conformation ensemble restricted to a coarse-grained conformation defined by C^α^ and SC,^52^ includes (i) *U*_dynss_ disulfide bond formation potentials calculated over all permutations (nss)^53^ and (ii) U_SSS_ repulsion potential preventing the formation of triple disulfide bonds (eq 1).^44^ The first one was introduced in the UNRES model^46^ to allow simulations without interfering, by pointing in input parameters, as to which pair of cysteine residues are going to form a bond.^46^ Effectively, the potential allows disulfide bonds to form and break between any cysteine residues, whether such contacts are present in the native structure or not, hence allowing the study also of non-native disulfide bonds in the course of the simulation. The second is essential to maintain the correct character of disulfide bonds owing to the possibility of forming higher-order disulfide bonds caused by a highly flexible coarse-grained chain. The introduction of *U*_dynss_ and U_SSS_ allows the study of the influence of the formation and disruption of disulfide bonds in the course of the simulation. We used the UNRES force field where parameters were optimized to reproduce folding thermodynamics of the tryptophan cage and tryptophan zipper with an extension to treat the coupling between the backbone and the side-chain.^54^ In this study, the same version of the UNRES force field was used as in the previous investigation, in which the thermal stability of the RNase A was studied,^44^ allowing direct comparison of the results.
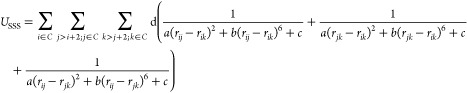
1where the *C* is the group of cysteine residues, *r* is the distance between the appropriate cysteines, and *a*, *b*, *c*, and *d* are user-defined parameters.

### Preparation of the System

For both coarse-grained and all-atom simulations, the RNase A experimental structure (PDB code: 1KF5),^55^ obtained at pH 7.1, was adapted as an initial conformation after the removal of a secondary set of atoms. Such a structure was then subjected to energy minimization (1000 steps in both models) and heated to 300 K (or 278 K).

### UNRES Coarse-Grained Simulations

In the UNRES model, only SMD simulations were performed, as conventional MD simulations were run in the previous paper,^44^ proving the stability of the RNase A. For each of the coarse-grained steered molecular dynamics simulations, 80 independent trajectories were run to obtain good sampling, as the coarse-grained model allows for more conformational flexibility and changes than the all-atom representation. CG simulations were performed with constant velocity^56^ pulling mode with a few different pulling speeds—0.0005, 0.001, 0.002, 0.004, 0.006, 0.008, 0.01, and 0.02 Å/4.89 fs (1 MTU)—to investigate the impact of pulling speed on the results. It corresponds to pulling speeds from approximately 0.01 to 0.4 m/s after taking into consideration that in the UNRES coarse-grained force fields events occur approximately 1000 times faster.^51^ The number of steps ranged between 1 000 000 and 15 000 000 with the time step set to 0.489 fs, performed to ensure a full stretch of the RNase A (over 500 Å); the time step was controlled with the use of the VTS algorithm.^50^ The spring constant was set to 1 kcal/mol/Å^2^. Langevin dynamics was used with friction scaled down by a factor of 100, as in previous works,^51^ to speed up the simulation, with the temperature of the thermostat set to 300 K. Snapshots and other information (such as forces) were saved every 200 steps.

The coarse-grained and all-atom simulations were performed in series that differ by the reductive/oxidative properties of the environment. Simulations without disulfide bonds did not require any additional treatment. In simulations with very high oxidative potential, static disulfide bond treatment was used, in which harmonic potential was applied like on any other covalent bonds in the UNRES model. For simulations with highly and weakly oxidative potential, the simulation environment was controlled by the use of dynamic disulfide bonds, which allowed for the formation and breaking of disulfide bonds during the course of the simulation. In the UNRES coarse-grained model, the depth of the disulfide bond formation potential controls the redox potential of the environment. It was set to 5.5 kcal/mol for weakly oxidizing and 11.0 kcal/mol for highly oxidizing environments. The second contact minimum was not affected by those changes, and only the bonded depth potential was changed; thus, the energy difference (energy barrier) between the formed and broken disulfide bond was effectively doubled.^46^ It should be noted that both values are in the range of experimental findings in different redox environments (approximately 4–22 kcal/mol).^57^

Owing to the use of dummy atoms capping protein termini in the UNRES model,^58^ the second and second to the last peptide groups (Glu2 and Ser123) were used as pulling anchors. Pulling by the second and second to the last residues instead of N- and C- termini should not have a qualitative impact on the results owing to low distance changes compared to the terminal residues and the rather unstructured character of the terminal parts of the RNase A.^59^

### Amber All-Atom Model and Simulations

Amber20 is a software package including tools to prepare, run, and analyze all-atom molecular dynamics with a wide selection of methods, force field parameters, and conditions.^60^ As the mechanical stability of the RNase A upon mechanical stretching would require a big enough periodic box to fit the whole protein without interbox contacts, it would require a box with a minimum dimension above 500 Å to fully stretch RNase A; therefore, running all-atom simulations with explicit water was not achievable in a reasonable time scale owing to computational limitations. The implicit solvent model with recommended ff14SBonlysc force field parameters and modified VdW radii (mbondi3 set) for proteins was chosen to run MD and SMD simulations with the generalized Born solvent model, as it is the most reliable Amber implicit solvent model available to date.^61,62^ No pressure scaling and no periodic boundaries were applied owing to the use of implicit conditions during simulations. First, conventional MD simulation of RNase A was run with the presence of all and no disulfide bonds in the structure for 1000 ns. Each simulation consisted of five independent trajectories to obtain reliable statistical averages. Then all-atom SMD simulations were performed with a constant force for 2.5 × 10^9^ steps (1.25 × 109 for simulation with predefined disulfide bonds) with a time step of 2 fs, at a pulling speed of 0.1, 1, and 10 Å/ns, which corresponds to 0.01, 0.1, and 1 m/s, respectively, each starting from the native conformation. Snapshots and other information (such as forces) were saved every 5000 and 200 steps, respectively. As in the coarse-grained simulations, Langevin dynamics was used with the temperature set to 300 K (or 278 K). Bonds involving hydrogen were constrained with the SHAKE algorithm to speed up the calculations and increase their stability. C^α^ atoms of terminal residues (Lys1 and Val124) were used as pulling anchors, which is a close comparison to conditions in the single-molecule AFM experiment.

Additionally, SMD simulations with restraints on the native disulfide bonds imposed were performed. The restraint energy tends to increase linearly at large values of distance.^60^ The restraints function is given by eq 2:
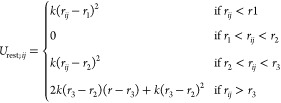
2where *U*_rest_;_*ij*_ is the restraints potential between the *i*th and *j*th center, *r*_*ij*_ distance between the *i*th and *j*th center, *k* is the spring constant, and *r*_1_, *r*_2_, and *r*_3_ are user-defined points of switching functions.

**Figure 2:**
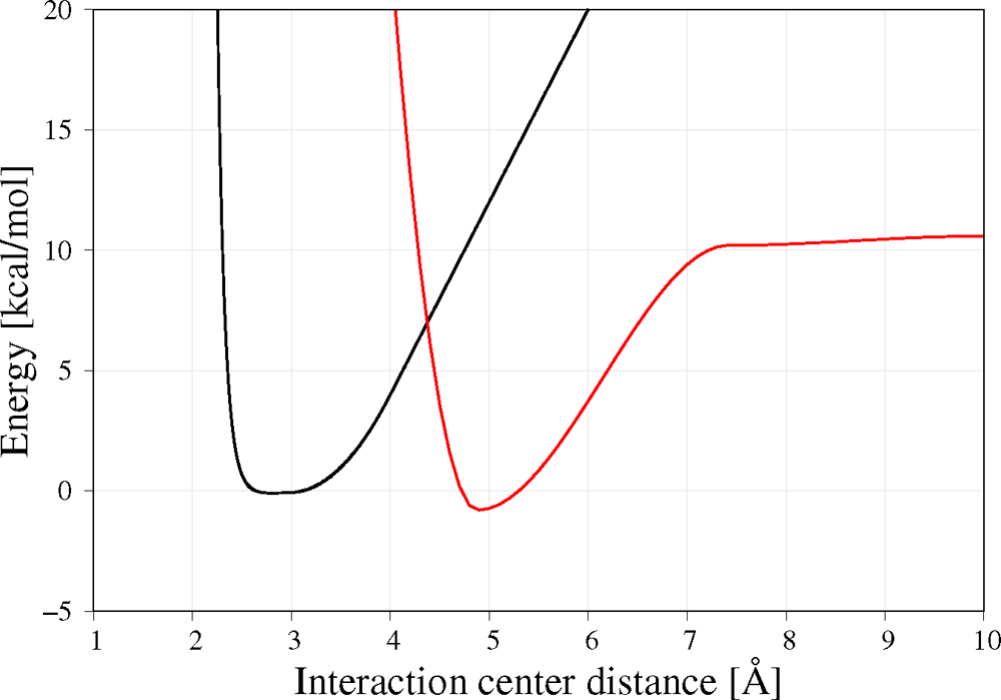
Comparison between dynamic disulfide bonds energy profile for the linear C^α^–SC···SC–C^α^ orientation (red) with an all-atom restraints energy profile, which includes the LJ potential from hydrogen and sulfur atoms (black).

The spring constant was set to 4.0 kcal/mol, and switching function distances *r*_1_, *r*_2_, and *r*_3_ were set to 1.8, 3.0, and 4.0 Å, respectively. Those values were adjusted by trial and error to mimic the UNRES disulfide bond energy profile, which is based on experimental findings (Figure 2) while being numerically stable.

Above the *r*_3_ value, the restraint energy depends linearly on the distance; therefore, the force acting on the system is constant and not distance-dependent. The constant force value equals 556 pN. In the case of SMD, the addition of the constant force should not affect the results significantly as the stretching spring is constantly moving, although it will be reflected as an additional force in the force against extension plots. Therefore, the force was subtracted from the results once the disulfide bond was broken.

### Data Analysis

Structural properties, such as RMSD, Rg, and RMSF, were analyzed for classical MD simulations. Root-mean-square deviation (RMSD), radius of gyration (Rg), and maximum radius of gyration (Rgmax) were calculated with the cpptraj for 1.0 μs of all-atom molecular dynamics. The root-mean-square fluctuation (RMSF) was also calculated with the use of the cpptraj but with the use of the second half of 1.0 μs conventional MD trajectories.

The fraction of the stable disulfide bond, both native and nonnative, was obtained by the calculating the distance of C^β^ atoms in cysteines that were involved in the bond, and the cutoff was set to 5.5 Å to check if the bond was present. The secondary structure was obtained with the use of the DSSP method of Kabsch and Sander^63^ built in the cpptraj. For the coarse-grained structures, the all-atom representation was rebuilt with the use of PULCHRA^64^ to reconstruct the backbone, SCWRL^65^ to reconstruct heavy atoms of the side-chains, and tleap to reconstruct the hydrogens. All the results obtained from the simulations were averaged over the RNase A extension with the bin size of 1 Å, and the standard mean error was calculated. To determine stable structure representatives during stretching, the ensemble of structures near the force (±1 Å) minima was extracted. For each conformation in the ensemble, the RMSD to each other was computed. The one with the lowest sum of the RMSD to other structures was selected as a representative. Visualization of results was done with the use of Gnuplot and Pymol.

## Results and Discussion

Structural properties, such as RMSD, Rg, and RMSF plots against time, were analyzed, revealing that RNase A is stable in the AMBER force field (total RMSD about 4 and 5 Å for simulation with all disulfide bonds present and absent, respectively; Figures S1 and S2). Rg plots demonstrate that the use of an implicit solvent causes a slight tendency to form a more compact structure (approximately 1 and 1.5 Å decrease of Rg and Rgmax, respectively). Interestingly, slightly more compact structures were observed for simulations without disulfide bonds, indicating that the protein without disulfide bonds can undergo more extensive conformational changes.

### Influence of the Temperature and Pulling Speed on SMD Results

Analysis of the SMD simulations run in various conditions (pulling speed and temperature) showed that SMD results are dependent on several factors. The exact peak position depends on the speed of pulling (Figure S3). The faster the pulling speed, the more shifted toward longer extensions the peak position is. This could be explained as the faster the pulling, the faster the system needs to adapt and the exterior changes (in this case, moving stretching spring); therefore, the system lags with the respect to pulling. An increase in the pulling speed causes the increase of the forces and relative peak sizes, which is commonly observed in SMD simulations.^52,66^ Additionally, owing to the lower amount of time that system has to adapt to the conformational changes force by applying an additional force, the differences in behavior between trajectories are larger in the case of faster pulling speeds, which caused larger observed SEM values. Extremely high speed (1 m/s) in all-atom simulation leads the peaks to be less sharp (Figure S3D), as the structure no longer has time to reform new secondary structures. The temperature of the simulation also influences the peak position because the lower the temperature, the slower the conformational changes. Therefore, the same effect for lower temperature as in the case of faster pulling is observed (Figure S4). Interestingly, the shape of the force against extension plots is not dependent on pulling speed, and apart from pulling speed 0.4 m/s for coarse-grained simulation and 1 m/s for all-atom simulation, all force-against-stretch plots bear high similarity to other pulling speeds (Figure S3). The slower the pulling, the larger the ensemble of conformation for a given extension obtained, which makes analysis more obscure and difficult, and the force peaks are less visible. A similar observation was done previously by the group of Klas Schulten that force constant impacts the magnitude of measured forces, but it does not affect the qualitative features of the process.^67^

For the above reasons, to fully see and analyze peaks and processes connected to their behavior, we focused mostly on the analysis of the ≈0.2 m/s pulling speed for coarse-grained simulations and the 0.1 m/s pulling speed for all-atom simulations.

### Influence of Native Disulfide Bonds on the RNase A Mechanical Stability

**Figure 3:**
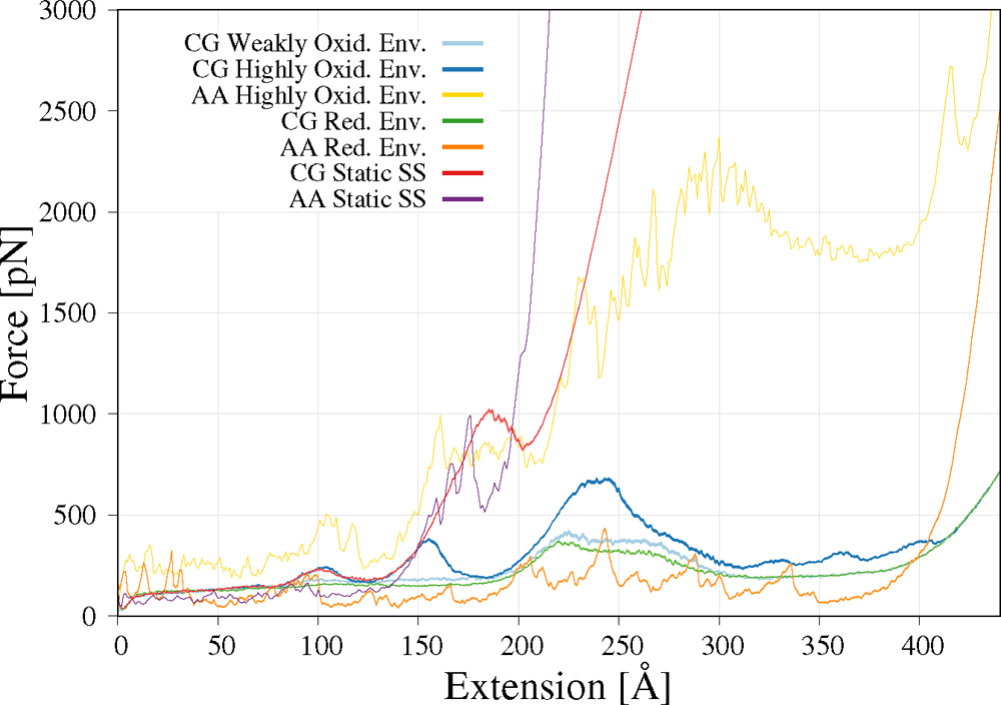
Average force against the RNase A extension from SMD simulations run with 0.1 and 0.2 m/s pulling speeds in all-atom and coarse-grained representations, respectively, with different disulfide bond treatments: reductive environment (no disulfide bonds present), static (disulfide bonds treated as unbreakable covalent bonds), and dynamic in weakly and highly oxidative environments.

As can be seen in Figure 3, for the static disulfide bonds, the force rises rapidly when the extension reaches about 120 Å for both all-atom and coarse-grained simulations because disulfide bonds cannot be broken and prevent RNase A from complete unfolding. An additional small force maximum can be observed for the all-atom simulation at the extension of about 90 Å and the coarse-grained simulation at the extension of about 100 Å. This local maximum aligns well with the maximum obtained for the SMD coarse-grained simulations in a highly oxidative environment.

For the coarse-grained simulation in a highly oxidative environment, three distinctive maxima are observed at the approximately 100, 150, and 230 Å extensions. For the simulation in a weakly oxidizing environment and without disulfide bonds, two overlapping maxima can be seen (Figure 3). The first maximum is at the extension of approximately 220 Å and the second at the extension 270 Å. These results correspond partially to all-atom simulations without disulfide bonds, as three maxima corresponding to the extension region of 200–300 Å are present. In the coarse-grained simulations, the force peak is significantly wider than in the all-atom simulations. For the simulation in a weakly oxidizing environment and without disulfide bonds, two overlapping maxima can be seen (Figure 1). The first maximum is at the extension of approximately 220 Å and the second at the extension 270 Å. In the coarse-grained simulations, the force peak is significantly wider than in the all-atom simulations. An additional two maxima with extensions 80 and 320 Å are observed in all-atom simulations.

**Figure 4:**
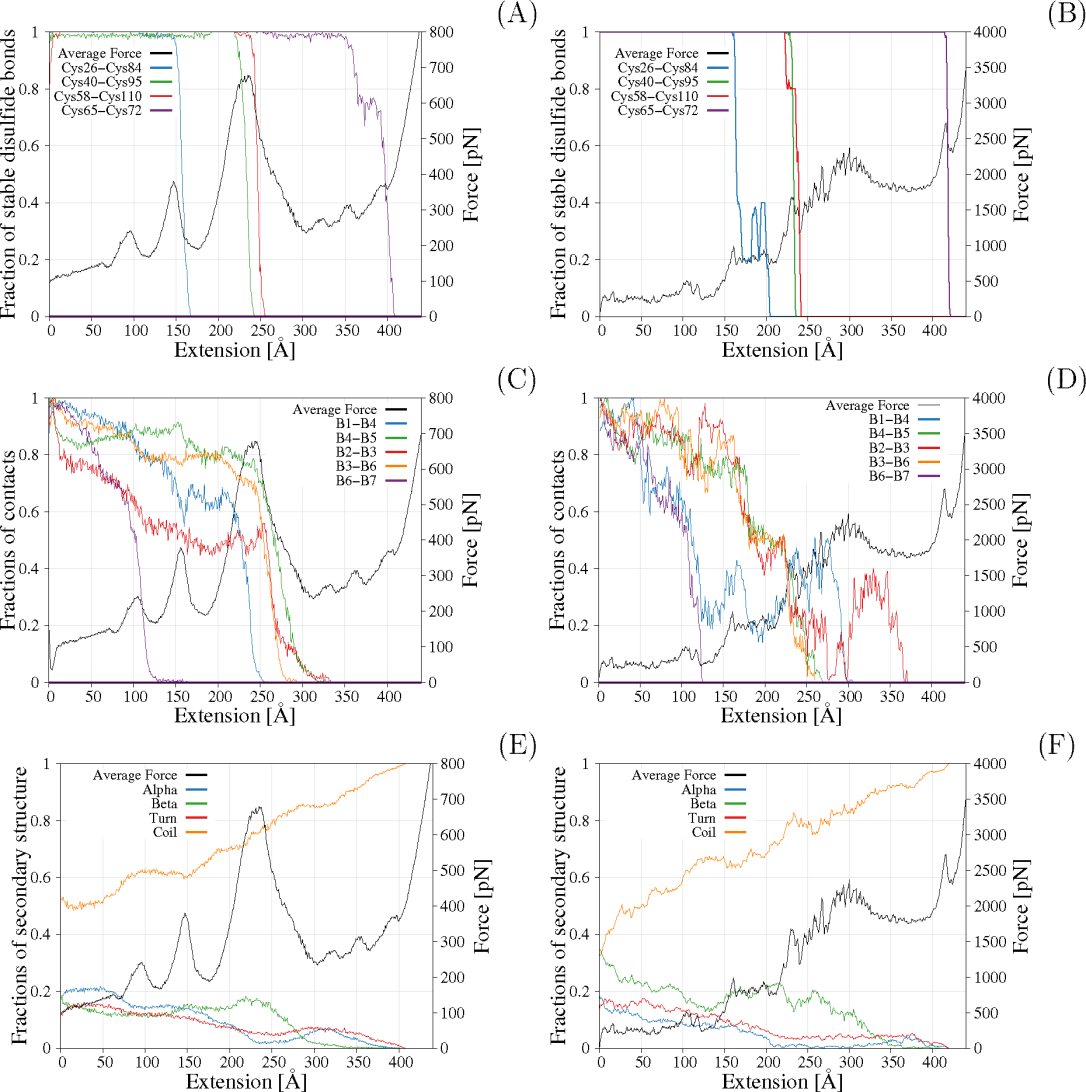
Comparison of analysis for native disulfide bonds (A, B), β-sheet fractions (C, D), and secondary structure (E, F) for coarse-grained simulation in the highly oxidation environment (A, C, E) and all-atom simulations with restraints (B, D, F) for SMD simulations run with 0.1 and 0.2 m/s pulling speeds in all-atom and coarse-grained representations, respectively.

When the disulfide bond’s influence on the mechanical stability is analyzed, it reveals a significant impact on the RNase A stability (Figures S5 and S6 and Figure 4A and B). The case of the reductive environment (Figure S5A and B) reveals that the contacts between cysteines disappear quickly and have virtually no impact on the stability of the protein. With weak oxidative conditions (Figure S5C), the situation is more complicated. In the case of Cys26–Cys84, there is virtually no influence on the structural stability as this disulfide bond is broken earlier than any significant force peak occurs. Similarly, for Cys65–Cys72 the disulfide bond is broken significantly later in terms of extension than the main force peak, and no peak force can be observed. However, this disulfide bond is the longest preserved in terms of extension. There is a loss of disulfide bond fraction associated with force peak in the extension at approximately 220 Å. The Cys40–Cys95 and the Cys58–Cys110 disulfide bonds are mainly broken during the force arising at the extension 220 Å. However, the loss of a fraction of those disulfide bonds starts significantly earlier. It seems rather that the influence of those disulfide bonds on the stability is small (if any), and the peak force is associated with the loss of secondary structure rather than the breaking of the disulfide bond.

A completely different picture emerges when the environment is highly oxidizing: in such conditions, disulfide bonds are very stabilizing fragments of the RNase A (Figure 4A and B). Each sharp loss of the fraction of the disulfide bond is associated with the force peak. The breaking order (in terms of extension) is Cys26–Cys84, Cys40–Cys95, Cys58–Cys110, and Cys65–Cys72 for both all-atom and coarse-grained simulations. The order of breaking during stretching is different from the thermal stability, where the Cys40–Cys95 bond is the least stable, while the Cys58–Cys110 and the Cys65–Cys72 are the most stable.^44^ This is also different from the folding pathway where the formation of the Cys40–Cys95 or Cys65–Cys72 needs to be the last to be formed.^68,69^ The loss of Cys65–Cys72 is associated with a small force peak, but Cys26–Cys84, Cys40–Cys95, and Cys58–Cys110 are associated with large force values. However, the Cys40–Cys95 is broken before the main force peak. This is consistent with the experimentally determined Cys26–Cys84 and Cys58–Cys110 structure stability enhancing properties.^70^ The breaking of Cys40–Cys95, Cys58–Cys110, and Cys65–Cys72 disulfide bonds in all-atom simulation occurs in single but separate events (Figure 4B), while a fraction of Cys26–Cys84 drops several times before completely disappearing. This indicates that there is more than one breaking pathway for the Cys26–Cys84 disulfide bond. The breaking of the first three disulfide bonds occurs in single but separate events (there is one pathway of breaking) in coarse-grained simulations. The breaking of the Cys65–Cys72 can be simultaneous with the breaking of the non-native Cys84–Cys95 or can occur after the breaking of this non-native disulfide bond. This pathway could not be observed in the all-atom force field as the non-native bonds cannot be formed there. After Cys65–Cys72 breaking, the structure is fully extended, and no further stable structure is observed. In general, a very similar behavior of breaking order of native disulfide bonds can be seen in both coarse-grained and all-atom simulations in a heavily oxidative environment, as well as the association of the disulfide bond breaking with significant force peaks.

### Influence of Non-native Disulfide Bonds on the RNase A Mechanical Stability

As non-native disulfide bonds can form only in the coarse-grained UNRES model, only these results are evaluated in this section. During the simulations, several disulfide bonds (or contacts between cysteines in a reductive environment) can be observed (Figure S5). The most significant non-native bond formed during the simulations is between Cys84 and Cys95. Contact between these two residues is observed in all types of simulations (except for predefined disulfide bonds). This interaction occurs in later stages of simulations and is associated with the main force peak, and in the case of the highly oxidative environment can lead to additional stability of RNase A (reflected in an additional force peak; Figure S6). Such behavior of maximizing the stability of the molecules by forming non-native contacts is widely observed in different types of molecules.^52^

### Loss of Secondary Structure Content during the RNase A Stretching

To determine which fragments of RNase A are the most stabilizing, the loss of a secondary structure against extension analysis was performed (Figures S7 and S8 and Figure 4C and D). In neither of the cases was the loss of the secondary structure a fast and single event. However, in all simulations, β7−β6 was the first structure to unfold.

The other β-sheet-unfolding order is not so easy to determine. The second β-sheet to unfold in all-atom simulation in reduced conditions is β3−β6 followed by β1−β4, while in the coarse-grained simulations (in all redox conditions) the order is reversed. Those two secondary structures unfold almost at the same time. In the all-atom simulation, the loss of β-sheet content is not so clear-cut, as multiple reformations are observed (Figure 4D). However, β1−β4 structure loss seems to precede the β3−β6, but then β1-β4 is reformed and unfolds as the last but one β-sheet.

**Figure 5:**
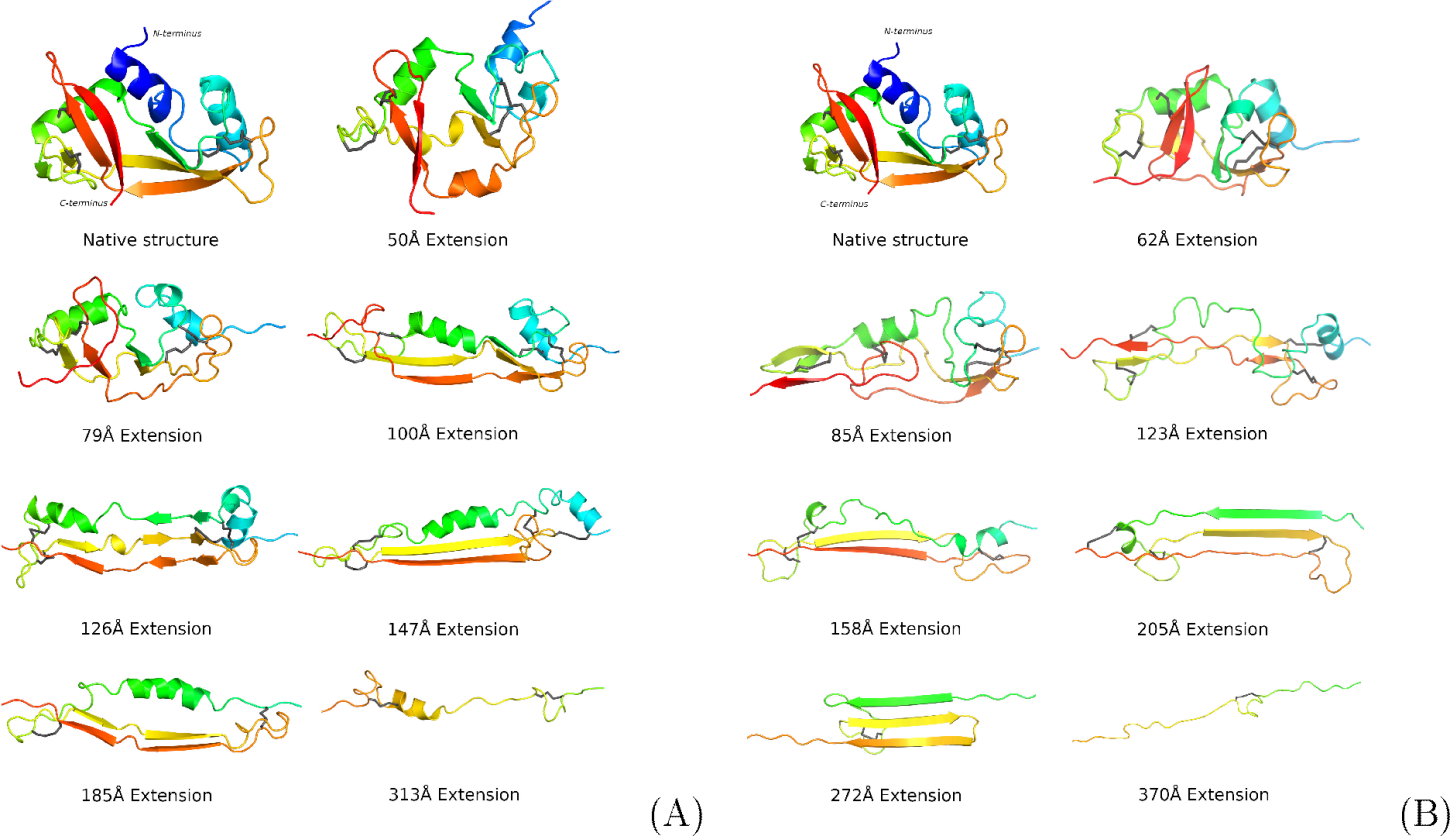
Cartoon representation of the semistable intermediate RNase A conformations obtained during coarse-grained stretching in the highly oxidative environment (A) and all-atom stretching with dynamic disulfide bonds (B) from SDM simulations run with 0.1 and 0.2 m/s pulling speeds in all-atom and coarse-grained representations, respectively. Fully extended fragments were removed from the figure for clarity.

The more oxidizing the environment, the longer in terms of extension is the β-sheet structure preserved. Moreover, stronger oxidative conditions lead to the more abrupt loss of β-sheet content as a function of extension. This further confirms the essential stabilizing role of disulfide bonds. Interestingly, the loss of β-sheet content preceded the increase in the distance between the sheets in all types of simulations. The first β-sheet to dissociate is β6−β7. The increase in distance between other β-sheets is associated with the main force peak in all simulations. The helical content steadily decreases throughout the stretching simulation with only two major events. The first is when the extension is approximately 60 Å in coarse-grained simulations or just at the very beginning of the simulation in all-atom simulations in a reduced environment. In this event, the first helix unfolds (Figure 5), followed by the distortion of the second helix (Figures S9–S11 and Figure 5A and B). The second event is associated with the main force peak when almost all the helical content is lost (though regained after some time/extension). The complete helical loss in all-atom simulations with restraints is shifted to the first force peak. The regaining of the helical content is observed in all types of simulations (Figures S9–S11 and Figure 4E and F), although the location of the helix is different than in the native structure. In general, all-atom simulations confirmed this order of events, with a small deviation of higher β-to-α ratio of the secondary structure. One of the reasons for that is the fact that coarse-grained simulations require conversion to all-atom models in order to calculate a secondary structure content using the DSSP algorithm, which may lead to small distortions, especially in the case of β-sheets, which are nonlocal, long-distance interactions of mostly side-chains. Such reconstruction imperfections especially explain the drop of β-content at the very beginning of the pulling in UNRES simulations. However, in the much later stages of stretching (150–200 Å), UNRES tends to form an α-helix and two β-strand/β-sheets (Figure 5A), while in Amber most dominating conformations contain three β-strands (Figure 5B). This is also observed as the reformation of the β2−β3 native-strand interactions, which is observed only in all-atom simulations around 300–360 Å of stretching.

In general, most unfolding events upon the application of mechanical tension to N- and C-termini start from unfolding N-terminal helices, with β6−β7 being the first structural part to unfold. These two processes are easily explained, as the terminal parts of a protein usually are the least resistant, and in this case, the external force is applied to these regions. However, the rest of the unfolding process does not follow that pattern, and the first disulfide bond to break is Cys26–84 and not Cys58–Cys110, which connects part of RNase A close to the pulling anchor (β6). Then almost simultaneous unfolding of β3−β6 and β1−β4 and disruption of Cys40-Cys95 appear, which is followed by disruption of all remaining β-sheets and Cys58–Cys110, the last to break being Cys65–Cys72, which is predated only by losing all helical content by the molecule.

## Conclusions

In this article, we performed all-atom and coarse-grained simulations of RNase A in various redox environments, which presented a very similar view of the protein stability. RNase A activity depends on the redox state,^71^ while our results revealed that the more oxidizing the environment, the more mechanically stable the RNase. Both our thermal unfolding and mechanical stretching studies show that the most stable disulfide bond is Cys65–Cys72. The least stable to mechanical stress is Cys40–Cys95, which is strongly connected to the local structure, in which cysteine residues forming these bonds are present. The two structural disulfide bonds Cys58–Cys110 and Cys26–Cys84 are associated with significant forces. In a highly oxidizing environment, the breaking of the disulfide bond correlates well with the force peak, while in the weakly oxidizing and reductive environment, there is no such correlation, showing that disulfide bonds have only a limited impact on stability. This indicates that the redox environment strongly determines the mechanical resistance of the proteins.

Unfolding of the stable secondary structure elements, such as β-sheets and α-helices, was not a single event and occurred gradually throughout the simulations. Moreover, during RNase A stretching, a quick reformation of the helices and β-sheets can also be observed. However, we have been able to identify the order of unfolding during the stretching, which started from the deformation of fragments closest to the termini: β6−β7 and N-terminal helix 1, followed by almost simultaneous unfolding of β3−β6, β1−β4, and the second and third helices. We noticed that in a highly oxidative environment, the presence of stable disulfide bonds stabilized secondary structure elements, this effect being especially visible for β-sheets. RNase A is a perfect example of how important it is for cells to control redox potential in their components and how it can impact biomacromolecules.

## Supporting Information Available

Diagrams of RMSD, RMSF, and Rg of RNase A in all-atom MD trajectories; diagrams of force upon stretching at different conditions; diagrams of fractions of disulfide bonds, secondary structure, and each of the β-sheets upon stretching; cartoon representation of the RNase A conformations obtained upon stretching (PDF)
