# Supplementary material for: Mechanical Stability of Ribonuclease A Heavily Depends on the Redox Environment
Source: J Phys Chem B. 2022 Aug 17;126(33):6240–9. doi: 10.1021/acs.jpcb.2c04718 (PMC9421896; doi:10.1021/acs.jpcb.2c04718)
Supplement: Supplementary file 1 — jp2c04718_si_001.pdf [file jp2c04718_si_001.pdf]

Supporting Information for "Mechanical Stability of Ribonuclease A Heavily Depends on the Redox Environment"

Pamela Smardz,[a] Adam K. Sieradzan,[b] Paweł Krupa[a]

[a]Institute of Physics Polish Academy of Sciences, Al. Lotników 32/46, 02-668 Warsaw, Poland
[b]Faculty of Chemistry, University of Gdańsk, Wita Stwosza 63, 80-308 Gdańsk, Poland

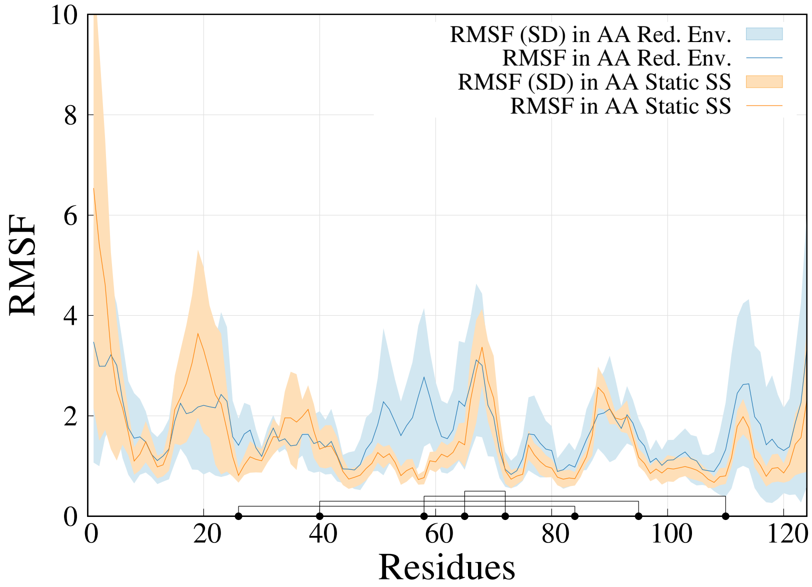

Fig. S1: Root-mean-square fluctuations (RMSF) of each C α atom, for canonical all-atom simulations without (solid blue line) and disulfide bonds (solid orange line). Cysteine residues (black dots) involved in disulfide bridges are marked with black lines.

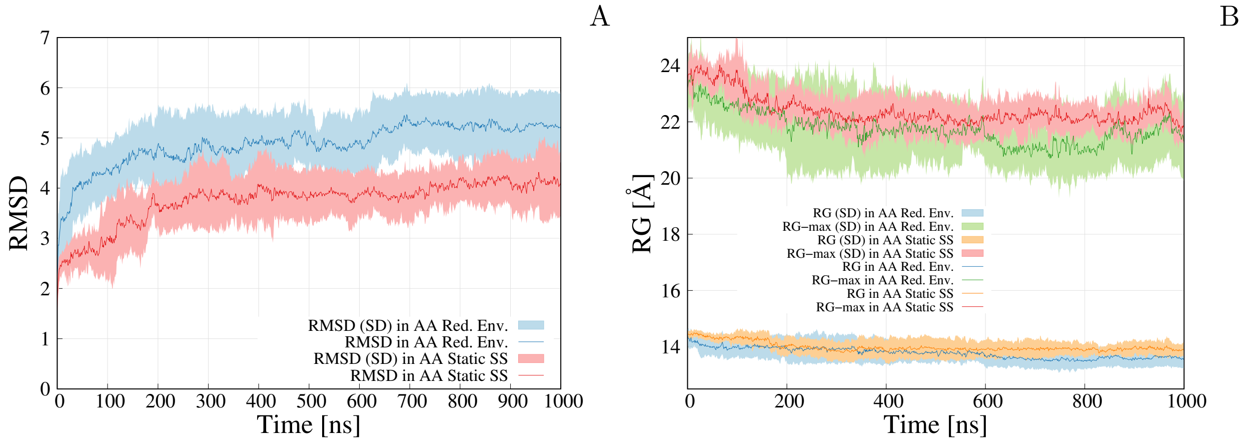

Fig. S2: RMSD (A) and plot of RG and RG max (B) of each C α atom, for canonical all-atom simulations without (solid blue and green line) and disulfide bonds (solid red and orange line).

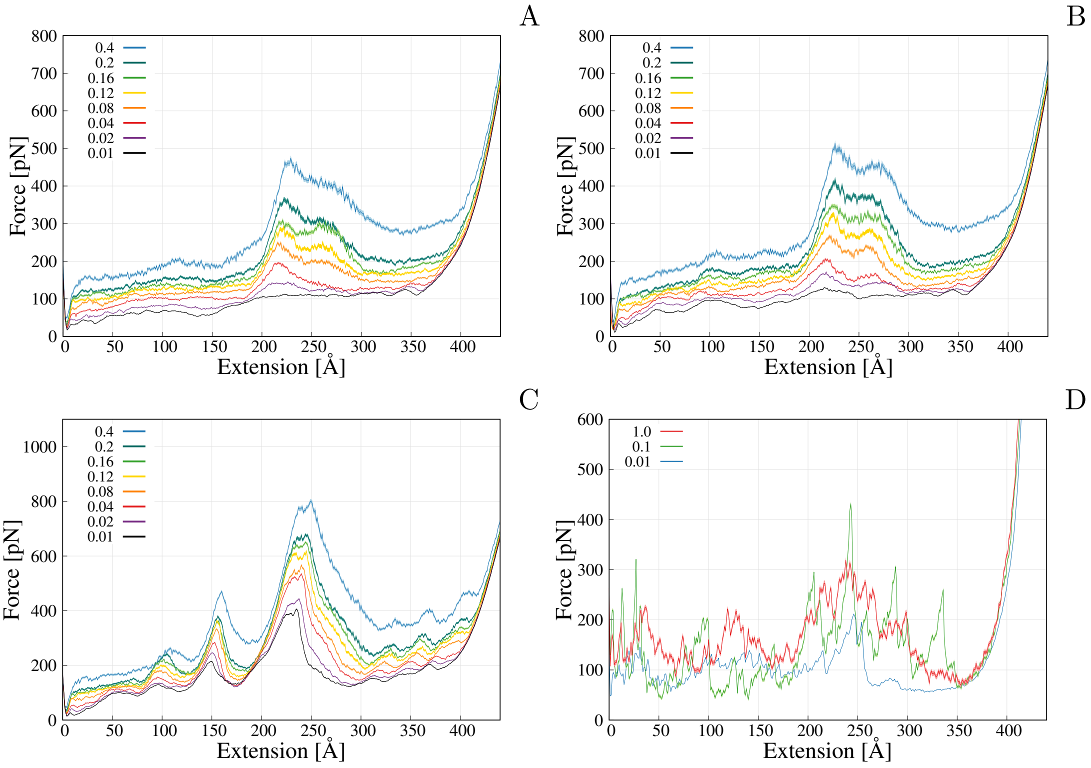

Fig. S3: Comparison of simulations in coarse-grained reductive (A), weak (B) and highly (C) oxidativ enviroment of different velocities from 0.04 to 0.4 m/s at 300K, and in reductive enviroment for different velocities in all-atom simulations (D) from 0.01 to 1 m/s at 300K.

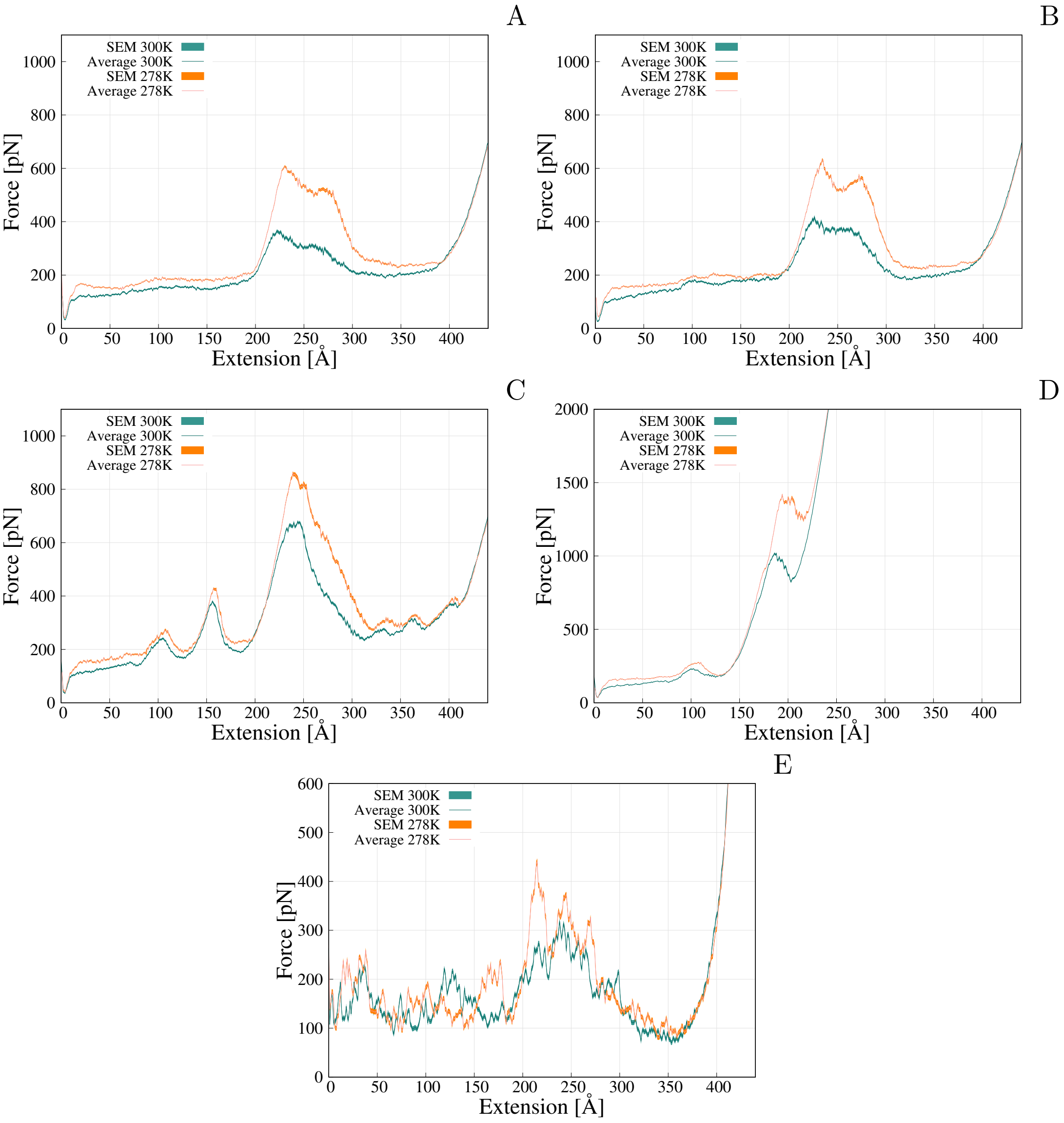

Fig. S4: Comparison of simulations in coarse-grained reductive (A), weak (B), and highly oxidative (C) enviroment and simulations with static disulfide bond (D) for velocity 0.2 m/s, and in all-atom representation for velocity 1 m/s (E) at temperatures 278K and 300K.

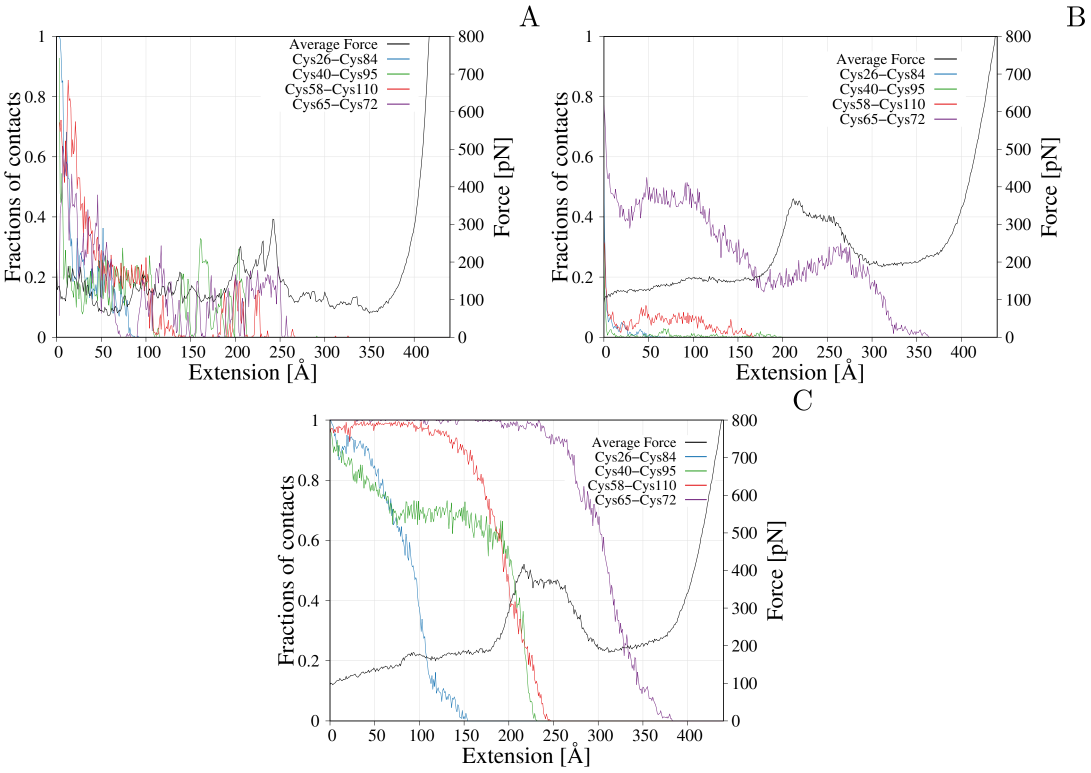

Fig. S5: Fraction of contacts between Cys residues forming native disulfide bonds in all-atom simulation without predefined disulfide bonds at 300K (A), and fraction of native disulfide bonds in coarse-grained simulation in reductive (B) and weak (C) oxidation enviroment at 300K.

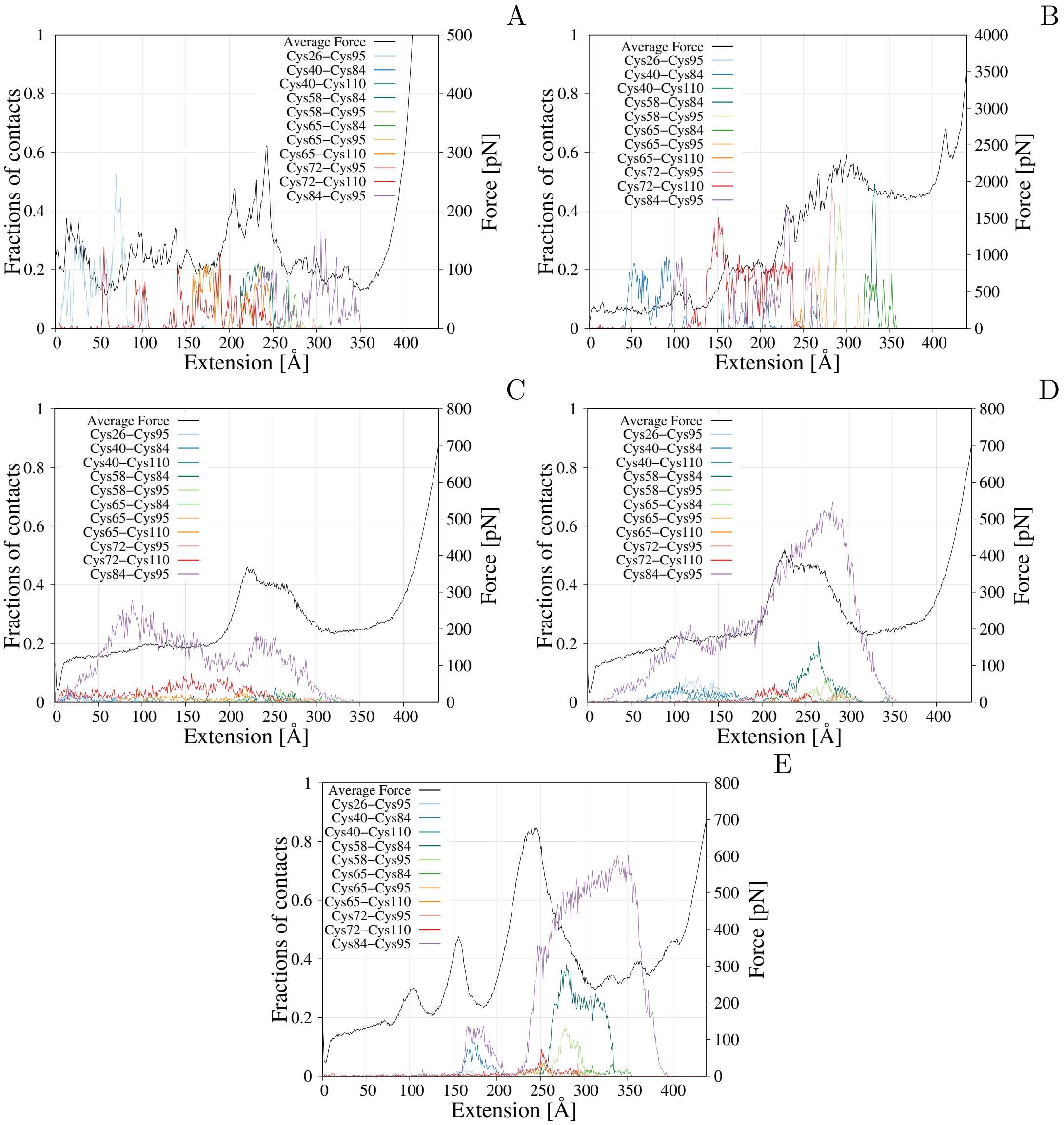

Fig. S6: Fraction of nonnative disulfide bonds in all-atom simulation without (A) and with disulfide bonds restraints (B), and in coarse-grained simulation in reductive (C), weak (D) and highly (E) oxidative enviroment at 300K.

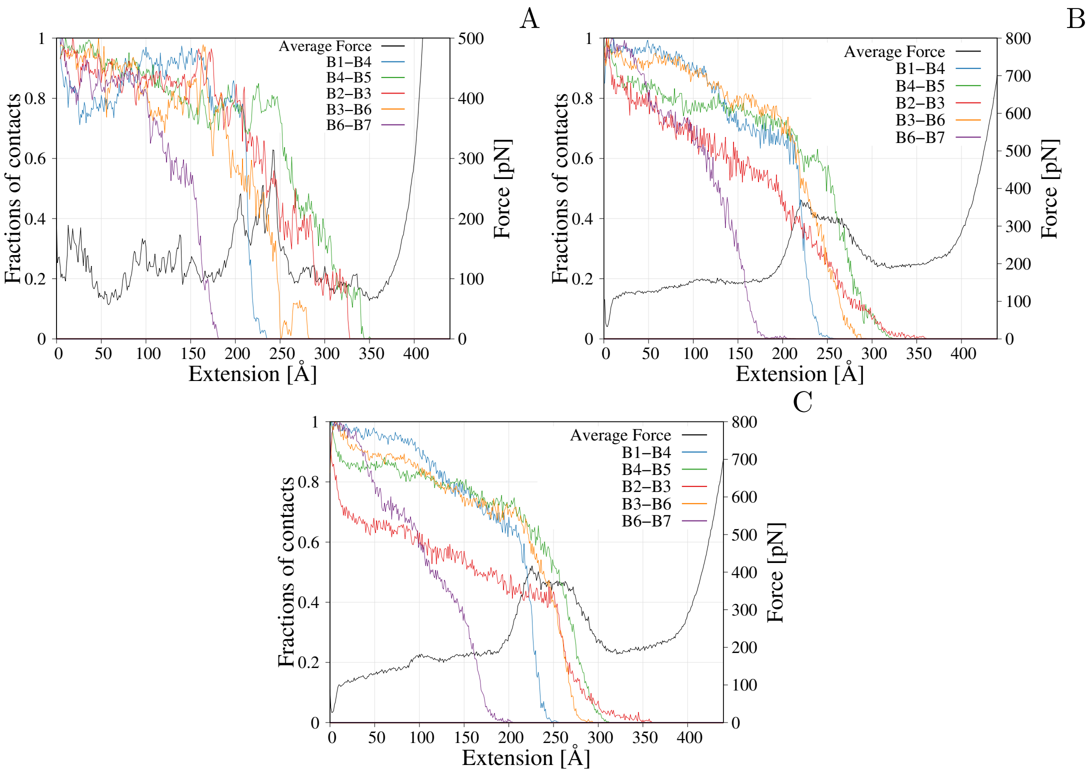

Fig. S7: Fraction of beta sheets in all-atom simulation without disulfide bonds (A) and fraction of beta sheets in coarse-grained simulation in reductive (B) and weak oxidation (C) enviroment at 300K.

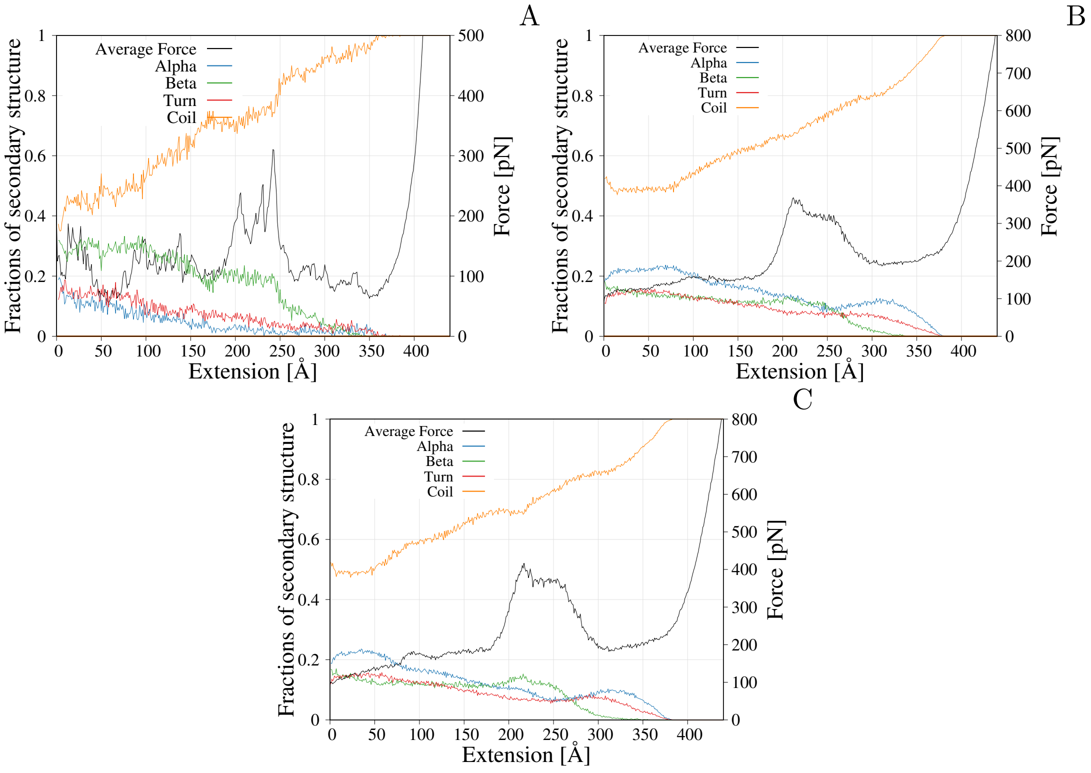

Fig. S8: Fraction of secondary structure in all-atom simulation without disulfide bonds (A), in coarse-grained simulation without disulfide bonds (B), and in weak (C) oxidizing environment at 300K.

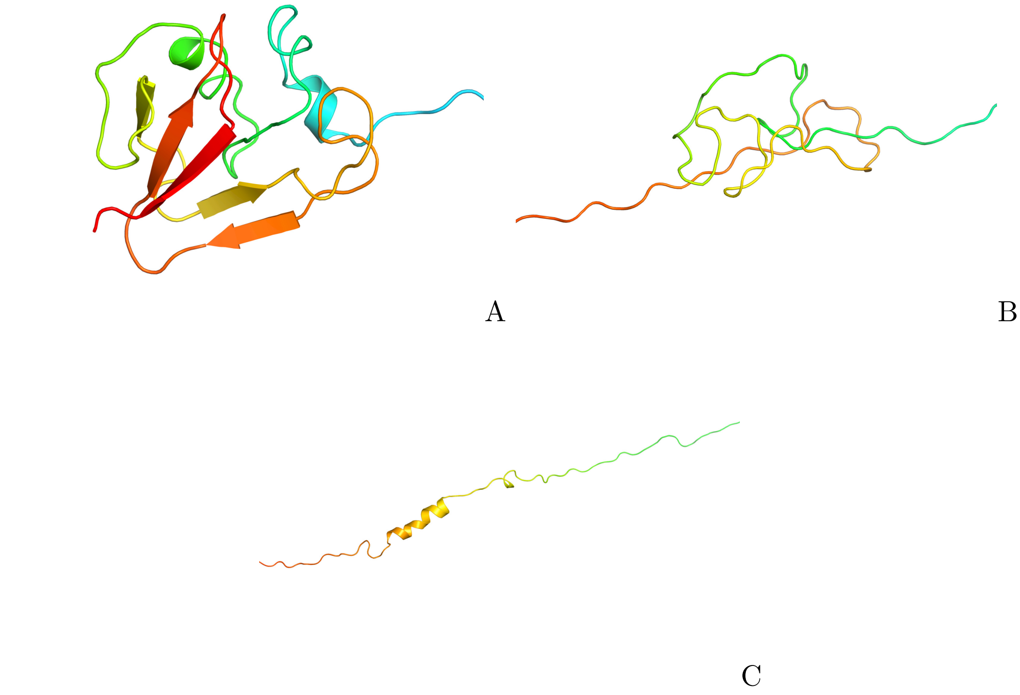

Fig. S9: Cartoon representation of the stable structures (for extensions: 68Å (A), 189Å (B), 272Å (C)) of RNase A otained during stretching in all-atom simulations without disulfide bonds.

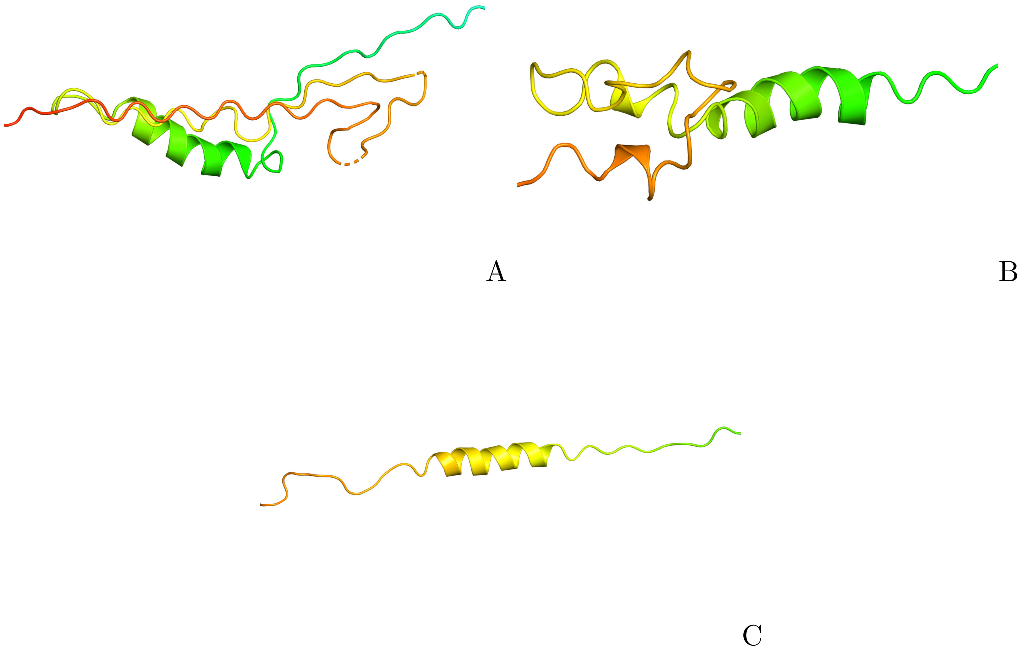

Fig. S10: Cartoon representation of the stable structures (for extensions: 174Å (A), 247Å (B), 317Å (C)) of RNase A otained during stretching in coarse-grained simulations without disulfide bonds.

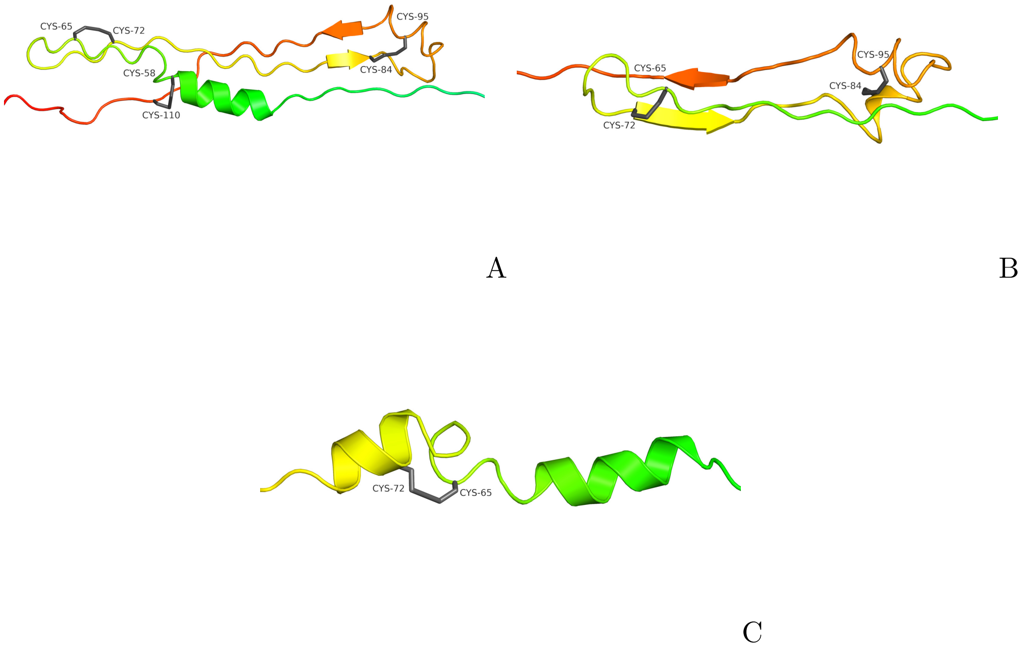

Fig. S11: Cartoon representation of the stable structures (for extensions: 180Å (A), 247Å (B), 327Å (C)) of RNase A otained during stretching in coarse-grained simulations in weak oxidizing environemnt.
